# Tribological Properties and 3D Topographic Parameters of Hard Turned and Ground Surfaces

**DOI:** 10.3390/ma15072505

**Published:** 2022-03-29

**Authors:** Viktor Molnar

**Affiliations:** Institute of Manufacturing Science, University of Miskolc, H-3515 Miskolc-Egyetemvaros, Hungary; viktor.molnar@uni-miskolc.hu

**Keywords:** tribology, hard turning, grinding, surface topography

## Abstract

Precision machining of automotive industrial parts is a highlighted topic in mechanical engineering due to the increased need for efficient and high-quality machining processes. This study is aimed to contribute to the field of surface topography evaluation by analyzing tribology-related topography parameters parallelly and finding connections between them. Hard machining experiments were carried out for the widely applied case-hardened material 16MnCr5 and the 3D topography of the machined surfaces was measured and analyzed. Based on a comprehensive design of experiments cubic response functions were determined for the analyzed parameters and the coefficients of determination were calculated. It was found that the cubic response function is reliable for predicting the topography parameter values and there are strong relationships between counterpart parameters under certain circumstances The findings could help clarify the roles of the analyzed parameters in some tribological properties within the analyzed cutting circumstances.

## 1. Introduction

In the automotive industry several types of parts frequently require the application of precision machining to ensure relatively high geometric accuracy [1,2] and surface quality. The surface quality has high importance in functional surfaces and the characterization of contacted surfaces is essential in studying tribological problems, such as friction or wear [3,4]. The focus of this study is the functional surfaces which move relative to other surfaces, and within that the analysis of the topography parameters that determine tribological properties, namely the wear resistance and the fluid-retention ability [5,6]. Analysis of functional surfaces is important because the characteristics of surface topography [7,8] have a strong influence on the life of machined parts [9].

Several studies have found that 3D surface topography parameters provide more exact information about the surface quality than the 2D parameters, e.g., [10,11] and this statement was recently confirmed by studies carried out typically for their comparison [12,13]. In the present study the topography measurements were carried out by optical equipment, whose reliability is higher than that of the conventional stylus solution [14]. The analyses of this study were carried out for 3D surface topography parameters, namely the maximum peak height (*Sp*), the maximum valley depth (*Sv*), the peak material volume (*Vmp*), the valley void volume (*Vvv*), the skewness (*Ssk*) and the kurtosis (*Sku*). For complex topography characterization it is necessary to use several parameters in parallel [15,16].

There are cutting technologies that typically remove the surface peaks, which results in plateau-like surfaces [17,18], and consequently in decreased material volume in the peak zone of the surface and increased wear resistance [19]. The *Sp* and *Sv* parameters provide information about the wear resistance and fluid-retention ability, respectively. A lower maximum peak height results in higher wear resistance and a higher valley depth in higher fluid-retention ability [20]. Another parameter is the *Sp*/*Sz* ratio, which is called the emptiness coefficient. If this value is lower, the wear resistance is higher [21]. The so-called volume parameters are used for the characterization of the tribological properties of the peak and valley zones [22]. Earlier the Abbott-Firestone curve-based *Sk* analysis (parameter group) was used widely. The *Sa*1 and *Sa*2 parameters of this group provide information about the peak and valley volumes [23]. Recently, *Vmp* and *Vvv* are used. They are exact 3D volume parameters that measure the peak material volume and the valley void volume precisely. Lower *Vmp* values indicate higher wear resistance and higher *Vvv* indicates higher fluid-retention ability [19].

Plateau-like topography has fewer peaks, which results in lower friction. Such surfaces show better wear resistance. The *Ssk* value of a plateau-like and therefore wear-resistant surface is low or negative [24,25,26]. It was found that a negative *Ssk* with high *Sku* results in better wear resistance [27] and better fluid-retention ability [28] because this combination of the two parameters results in a low friction coefficient and facilitates lubricant distribution. The reason for this phenomenon is that a surface with such skewness and kurtosis values has sharp valleys that act similar to nanoscale reservoirs for the lubricant [29]. It was also reported that low *Ssk* indicates better fluid-retention ability but the *Sku* value does not influence it [30], although. Another study found that the fluid retention ability increases with the decrease of *Sku* [31]. These uncertainties led to the aim to analyze the skewness and kurtosis parameters too in this study.

The *Sp* and *Vmp* parameters inform us about the tribological characteristics of the peak zone of the surface and the *Sv* and *Vvv* about those of the valley zone. Although the dimension of the *Sp* is different from that of the *Vmp* (height and volume parameters, respectively), correlations can be observed among their values, because they express identical properties. The same is valid for the *Sv* and *Vvv* parameters. This hypothesis is tested in this study by the using the coefficient of determination, which has been used in other studies in this field [32,33].

Cutting experiments and 3D topography measurement of the machined surfaces were carried out to analyze the connections between some tribology-related topography parameters. In this paper two procedures—hard turning and grinding—were compared. They are standard precision machining procedures of automotive industrial parts, due to their advantageous surface integrity characteristics [34]. In the experiments the cutting data were varied to analyze their effects on the considered topography parameters and response function were determined for each parameter. The strengths of the connections among the cutting data and the topography parameters were expressed by the coefficient of determination. Two widely used precision machining procedures—hard turning and grinding—were compared based on experiments carried out by design of experiment, in which the main cutting data were varied. The novelty of the study is that a statistics-based method was applied for the evaluation of the strength between the analyzed parameters and a detailed experiment was carried out for determining relatively precise response functions for predicting the topography values based on cutting data.

The findings contribute to practical applications or academic studies: the relationships between the analyzed parameters are described based on a detailed experiment and these relationships are quantified.

## 2. Materials and Methods

**Figure 1:**
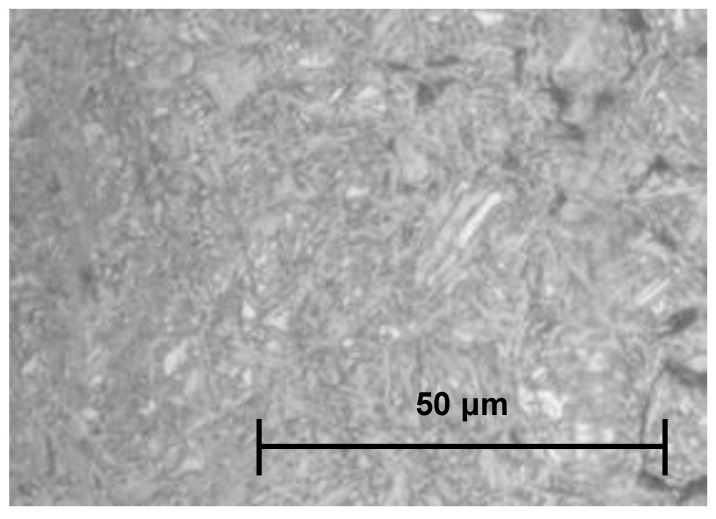
Microstructure of the used material (zoom: 500×).

The machined material was 16MnCr5. It is widely applied in the automotive industry, e.g., in machining gear wheels. The material can be case hardened, and it is mainly used for machining precision parts. The carburizing was carried out at 900 °C for 8 h. The temperature of case hardening was 820 °C and its duration was 30 min. For the cooling oil was used. The microstructure is demonstrated in Figure 1. The achieved hardness is 62–63 HRC and the microstructure is martensitic. The surface was etched in 2% nitric acid and was polished. A Zeiss Axio Observer D1m type microscope was used for the microstructure analysis. The hardness reached in present experiment requires a machine tool whose structure is rigid enough to avoid harmful vibrations when machining by single-point tools. The mechanical and physical properties and the chemical composition of the material are summarized in Table 1 and Table 2, respectively.

Hardness measurement was carried out on the workpieces before machining using a universal hardness tester type Reicherter UH250 (Buehler, Leinfelden-Echterdingen, Germany). The dwell time was 10 s, the load was 98.07 N. The data were processed by the software WIN-Control v. 2.98.9. For the measurement the standard DIN EN ISO 18,265 was applied. The HV10 tests were repeated five times per workpiece. In Table 3 the results of the Vickers hardness test are summarized. The averages varied between 749 and 781 HV, and therefore the Rockwell hardness varied between 62 and 63 HRC. The relative deviations of the measurements were calculated, and they varied between 2.01 and 3.75. This means that the measurements can be considered as acceptable.

The machining experiments were carried out on a CNC lathe type Optiturn S600 (Optimum, Budapest, Hungary), which is capable of turning hardened surfaces, and on a universal cylindrical grinder type KE 250-04. The type of CBN insert used for hard turning was CNGA 120408TA4 and the tool holder was CLNR 2525M12. The used wheel for grinding was a ceramic bound alumina wheel type KA32M5KE. Its external diameter was 400 mm and its width 63 mm. In hard turning the cutting speed (*v*_c_), the depth-of-cut (*a*_p_) and the feed rate (*f*) were varied; in grinding the infeed velocity (*v*_fR_) and the revolution-per-minute (*n*) of the workpiece were varied. The technological data were set to four levels within the range recommended by the tool manufacturers for the tool and workpiece material pair. The design of experiment is summarized in Table 4 and Table 5. The setups for hard turning are designated by the letter ‘T’ and for grinding by ‘G’. The machining experiments were carried out on eight workpieces. The surfaces hard turned at *a*_p_ = 0.05 mm, were used in the next step for grinding.

For the roughness measurement a 3D roughness tester machine type AltiSurf 520 (Altimet, France) was used. The type of the optical sensor was CL2. For the evaluation of the data *λ*_c_ = 0.8 mm cut-off and Gauss filter were applied. The x- and y-direction resolutions were 2 μm, the z-direction resolution was 0.012 μm. The measurement range in z-direction was 0–300 μm. For the analysis of the parameters the standard ISO 25,178 was used. The evaluation area was 2.45 mm × 2.45 mm.

**Figure 2:**
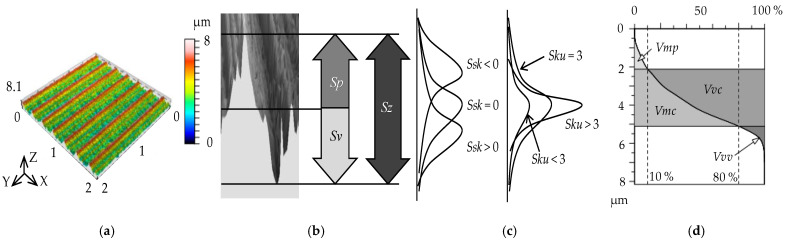
(**a**) The topography of a periodic surface, the determination of the (**b**) maximum peak height (*Sp*), maximum valley depth (*Sv*), maximum height (*Sz*), (**c**) skewness (*Ssk*), kurtosis (*Sku*), (**d**) peak material volume (*Vmp*) and the valley void volume (*Vvv*) parameters.

In Equations (1)–(4) the applied parameters (arithmetic mean height—*Sa*, maximum height—*Sz*, maximum peak height—*Sp*, maximum valley depth—*Sv*, skewness—*Ssk* and kurtosis—*Sku*) are defined. In Figure 2 the visual interpretations of these and the definition of the peak material volume—*Vmp* and the valley void volume—*Vvv* are demonstrated. In Figure 2a the 3D photographic view of a periodic surface, in Figure 2b the maximum peak height (*Sp*) and valley depth (*Sv*) and the sum of them, the maximum height (*Sz*), in Figure 2c the interpretation of the skewness (*Ssk*) and the kurtosis (*Sku*) and in Figure 2d among other volume parameters the interpretation of the peak material volume (*Vmp*) and the valley void volume (*Vvv*) are demonstrated. The *Sp* and the *Sv* are measured from the center plane of the profile to the highest peak and valley points, respectively. The *Sz* is the total height of the profile. The *Ssk* provides information about the degree of bias of the asperity (how asymmetric the distribution of the surface points), the *Sku* is the measure of the sharpness of the surface. The *Vmp* represents the volume of material at material ratio 10%, the *Vvv* represents the dale at the material ratio 80%.

(1) $$Sa=\frac{1}{A}\overset{}{\underset{A}{\iint }}\left|Z\left(x,\;y\right)\right|dx\;dy$$

(2) $$Sz=Sp+Sv=\underset{A}{\max}Z\left(x,\;y\right)+\left|\underset{A}{\min}Z\left(x,\;y\right)\right|$$

(3) $$Ssk=\frac{1}{Sq^{3}}\left[\frac{1}{A}\iint_{A}Z^{3}\left(x,\;y\right)dx\;dy\right]$$

(4) $$Sku=\frac{1}{Sq^{4}}\left[\frac{1}{A}\iint_{A}Z^{4}\left(x,\;y\right)dx\;dy\right]$$

In this study response functions were determined. These are regression functions, and their coefficients (**b**) can be estimated by the least square method. The general form of the function is:

(5) $$y^{\prime }=bX+\varepsilon$$

where **y** is the vector of the predicted values (roughness parameters), **X** is the matrix of the independent variables on all the experiment levels and **ε** is the residuum vector. Its values are not part of the response functions. The coefficient of determination informs about the goodness of the response function that predicts the real value. The **b** coefficients vector can be determined by:

(6) $$b={\left(X^{\prime }X\right)}^{-1}X^{\prime }y$$

where **X**′ is the adjugate of the **X** matrix and **y** is the vector of the dependent variable (measured roughness values).

## 3. Results and Discussion

### 3.1. Roughness Values and Response Functions

The arithmetic mean height (*Sa*) and the maximum height (*Sz*) characterize the surface roughness broadly. The 2D counterparts of these parameters (*Ra*, *Rz*) are widely used in part drawings; however, they provide only a little information about the characteristics of the surface topography and so about the suitability for the surface’s functional requirements.

**Figure 3:**
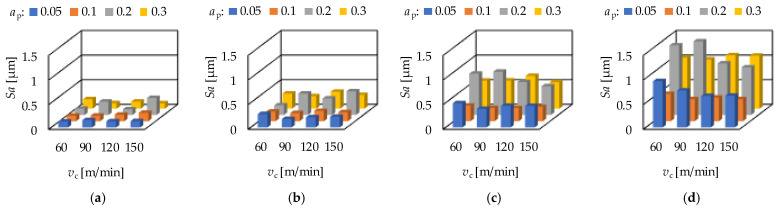
The measured arithmetic mean height (*Sa*) values for hard turning by applying (**a**) *f* = 0.05 mm/rev, (**b**) *f* = 0.1 mm = rev, (**c**) *f* = 0.15 mm/rev, (**d**) *f* = 0.2 mm/rev.

**Figure 4:**
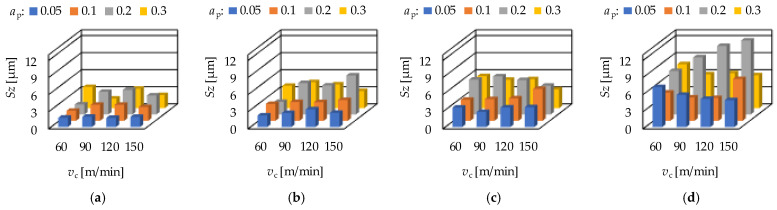
The measured maximum height (*Sz*) values for hard turning by applying (**a**) *f* = 0.05 mm/rev, (**b**) *f* = 0.1 mm = rev, (**c**) *f* = 0.15 mm/rev, (**d**) *f* = 0.2 mm/rev.

In Figure 3 the arithmetic mean height (*Sa*) values and in Figure 4 the maximum height (*Sz*) values of the hard turning operation are demonstrated for the different feeds. From the figures it can be seen; that the average *Sa* values belonging to certain depth-of-cut (*a*_p_) levels show a certain tendency. The lowest averages are obtained on the levels 0.05 and 0.1 mm (when *f* is 0.1 or higher, the lowest averages were obtained at the depth-of-cut-level 0.1 mm); the highest averages were obtained at the depth-of-cut level 0.2 mm. Increasing average *Sz* values can be observed when the order of the depth-of-cut is: 0.05; 0.1; 0.3; 0.2 mm. Concerning the effect of the cutting speed, no tendency was observed either in the *Sa* or in the *Sz* values. The values show increase or decrease or periodicity, without any observable rule. At the same time, it can be stated that both the *Sa* and the *Sz* values increase by the feed rate (*f*). This is valid not only for the averages. The numerical values of the analyzed parameters are summarized in Appendix A.

**Figure 5:**
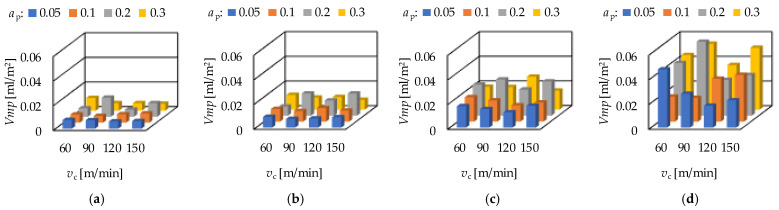
The measured peak material volume (*Vmp*) values for hard turning by applying (**a**) *f* = 0.05 mm/rev, (**b**) *f* = 0.1 mm = rev, (**c**) *f* = 0.15 mm/rev, (**d**) *f* = 0.2 mm/rev.

**Figure 6:**
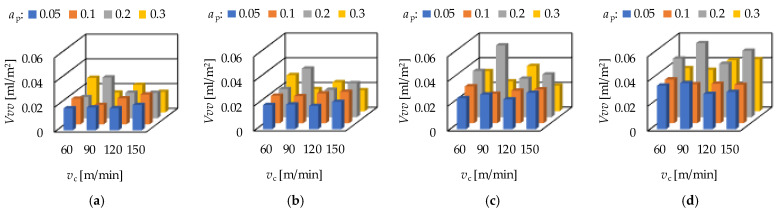
The measured valley void volume (*Vvv*) values for hard turning by applying (**a**) *f* = 0.05 mm/rev, (**b**) *f* = 0.1 mm = rev, (**c**) *f* = 0.15 mm/rev, (**d**) *f* = 0.2 mm/rev.

The peak material volume (*Vmp*) is in connection with the wear resistance of a surface. The valley void volume (*Vvv*) is in connection with the fluid-retention ability of a surface. Increasing average *Vmp* and *Vvv* values can be observed when the order of the depth-of-cut is: 0.05; 0.1; 0.3; 0.2 mm. There is one exception: *f* = 0.1 mm/rev, where the order of the depth-of-cut is 0.05; 0.3; 0.1; 0.2 mm. When varying the cutting speed, no tendency can be observed. Concerning the feed rate, the *Vmp* and *Vvv* values increase with the feed rate, which is valid not only for the averages. The *Vmp* and the *Vvv* values for hard turning are demonstrated in Figure 5 and Figure 6, respectively.

**Figure 7:**
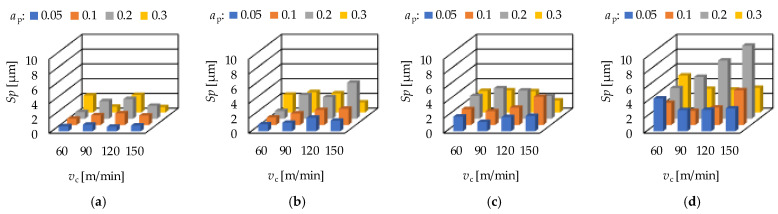
The measured maximum peak height (*Sp*) values for hard turning by applying (**a**) *f* = 0.05 mm/rev, (**b**) *f* = 0.1 mm = rev, (**c**) *f* = 0.15 mm/rev, (**d**) *f* = 0.2 mm/rev.

**Figure 8:**
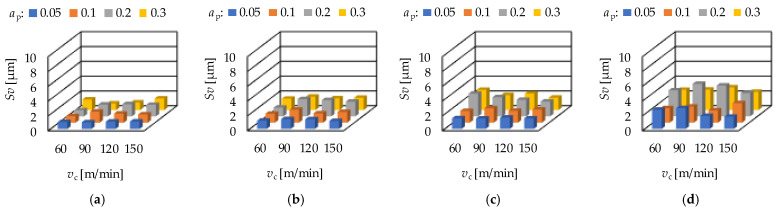
The measured maximum valley depth (*Sv*) values for hard turning by applying (**a**) *f* = 0.05 mm/rev, (**b**) *f* = 0.1 mm = rev, (**c**) *f* = 0.15 mm/rev, (**d**) *f* = 0.2 mm/rev.

The maximum peak height (*Sp*) is in connection with the wear resistance of a surface, while the maximum valley depth (*Sv*) is in connection with the fluid-retention ability. In hard turning the feed rate has a significant impact on these parameters. Both the *Sp* and the *Sv* values increase with the feed rate. Increasing average *Sp* and *Sv* values can be observed when the order of the depth-of-cut is: 0.05; 0.1; 0.3; 0.2 mm on the feed rates 0.05; 0.1 and 0.15 mm/rev. At the feed rate 0.2 mm/rev the order is: 0.1; 0.05; 0.3; 0.2 mm. The cutting speed has no clear effect on the *Sp* and *Sv* values. Concerning the feed rate, the *Sp* and *Sv* values increase with the feed rate, which is valid not only for the averages. The *Sp* and the *Sv* values for hard turning are demonstrated in Figure 7 and Figure 8, respectively.

**Figure 9:**
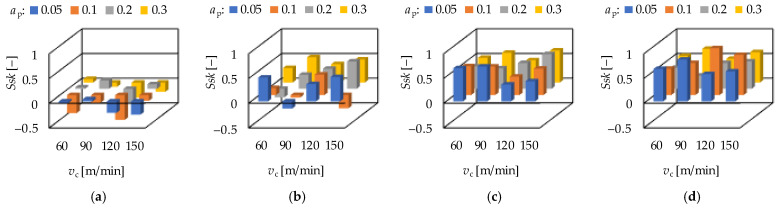
The measured skewness (*Ssk*) values for hard turning by applying (**a**) *f* = 0.05 mm/rev, (**b**) *f* = 0.1 mm = rev, (**c**) *f* = 0.15 mm/rev, (**d**) *f* = 0.2 mm/rev.

If the skewness (*Ssk*) value is zero or decreasing, both the wear resistance and the fluid-retention ability increase. Concerning the hard turning procedure, on lower feed rate lower *Ssk* values were obtained, relatively close to or below zero. There were no clear tendencies observed for the effects of the cutting speed or the depth-of-cut (Figure 9). The kurtosis (*Sku*) informs about the wear resistance.

**Figure 10:**
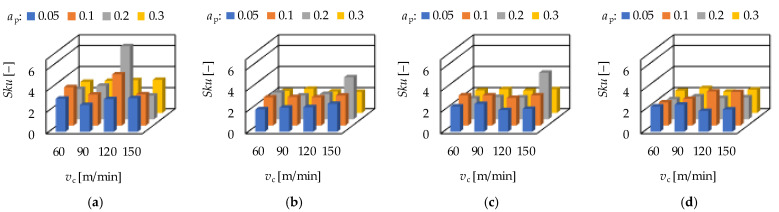
The measured kurtosis (*Sku*) values for hard turning by applying (**a**) *f* = 0.05 mm/rev, (**b**) *f* = 0.1 mm = rev, (**c**) *f* = 0.15 mm/rev, (**d**) *f* = 0.2 mm/rev.

If its value is lower 3 or decreasing, the wear resistance increases. By the increase of the feed rate lower *Sku* values were obtained. When varying the cutting speed on fixed ap and *f* levels or the depth-of-cut on fixed *v*_c_ and *f*, no clear tendencies were observed in the change of the *Sku* value (Figure 10).

**Figure 11:**
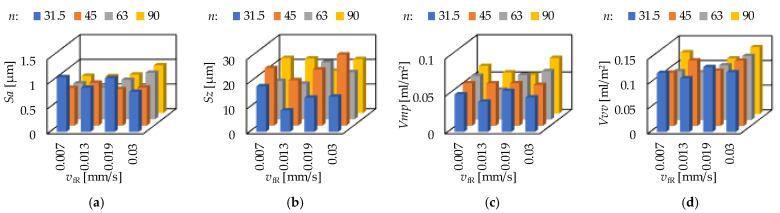
The measured (**a**) arithmetic mean height—*Sa*; (**b**) maximum height—*Sz*; (**c**) peak material volume—*Vmp* and (**d**) valley void volume—*Vvv* values for grinding.

**Figure 12:**
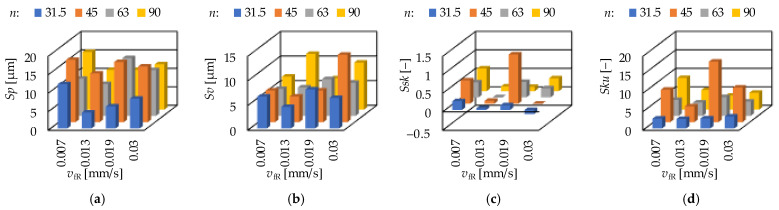
The measured (**a**) maximum peak height—*Sp*; (**b**) maximum valley depth—*Sv*; (**c**) skewness—*Ssk* and (**d**) kurtosis—*Sku* values for grinding.

The ground surfaces were machined by infeed grinding. The two influencing cutting data were the infeed velocity (*v*_fR_) and the rpm of the workpiece (*n*). The results are demonstrated in Figure 11 and Figure 12. It can be observed that there is no clear relationship between the cutting data and the topography parameters.

When the rpm is fixed at 31.5 1/min, the smallest arithmetic mean height (*Sa*) was obtained at 0.0302 mm/s. The smallest values of maximum height (*Sz*), peak material volume (*Vmp*), valley void volume (*Vvv*), maximum peak height (*Sp*) and maximum valley depth (*Sv*) were obtained at 0.0130 mm/s. When the rpm is fixed at 63 1/min, the smallest maximum valley depth (*Sv*) was obtained at 0.0069 mm/s. The smallest values of arithmetic mean height (*Sa*), maximum height (*Sz*), peak material volume (*Vmp*), valley void volume (*Vvv*) and maximum peak height (*Sp*) were obtained at 0.0130 mm/s. Concerning the other two rpm levels, no such clear tendencies were observed. A more detailed analysis for the strength of the influencing factors is necessary in the form of calculating the coefficients of determination.

### 3.2. Analysis of Roughness Determining Factors

The response functions of the analyzed roughness parameters were determined for both the hard turned and the ground surfaces. The coefficients of the independent variables (cutting parameters) in the response functions provide information about the extent and direction of how they influence the roughness parameter value. However, due to the different scales of the cutting parameters, the coefficient of determination provides more accurate information. The coefficient of determination (R^2^) informs us about the strength of the connection between the independent (e.g., *a*_p_, *v*_c_) and the dependent (e.g., *Sa*, *Sz*) variables, i.e., it shows what extent the former determines (explains) the latter in percentage form. The coefficient of determination is the square of the coefficient of correlation. The multifactorial coefficient of determination informs about the strength between the actual values and the values predicted by the response function. The connection is considered as extremely strong if 0.81 ≤ R^2^ < 1; strong, if 0.49 ≤ R^2^ < 0.81; medium, if 0.16 ≤ R^2^ < 0.49; weak, if 0.04 ≤ R^2^ < 0.16 and extremely weak, if R^2^ < 0.04.

Concerning the form of the response function, the quasilinear, the quadratic and the cubic forms were compared, and the highest multifactorial coefficient of determination was obtained in the case of the cubic form. The cubic forms for hard turning and grinding are demonstrated by Equations (7) and (8), respectively. The coefficients of the response functions are summarized in Table A6 and Table A7.

(7) $$\begin{array}{ll} y=b_{0}+b_{1}a_{p}+ & b_{2}v_{c}+b_{3}f+b_{11}a_{p}^{2}+b_{22}v_{c}^{2}+b_{33}f^{2}+b_{111}a_{p}^{3}+b_{222}v_{c}^{3}+b_{333}f^{3}+b_{12}a_{p}v_{c}+b_{13}a_{p}f\\ & +b_{23}v_{c}f+b_{112}a_{p}^{2}v_{c}+b_{113}a_{p}^{2}f+b_{221}v_{c}^{2}a_{p}+b_{223}v_{c}^{2}f+b_{332}f^{2}a_{p}+b_{331}f^{2}v_{c}\\ & +b_{123}a_{p}v_{c}f \end{array}$$

(8) $$y=b_{0}+b_{1}n+b_{2}v_{\mathrm{fR}}+b_{11}n^{2}+b_{22}v_{\mathrm{fR}}^{2}+b_{111}n^{3}+b_{222}v_{\mathrm{fR}}^{3}+b_{12}v_{\mathrm{fR}}n+b_{112}n^{2}v_{\mathrm{fR}}+b_{122}v_{\mathrm{fR}}^{2}n$$

In Table 6 the coefficients of determination for the analyzed procedures and roughness parameters are summarized. It can be stated that the feed rate is the strongest influencing factor of the roughness parameters in hard turning. The values of R^2^ varies between 0.201 and 0.650, i.e., the strengths of the relationships are strong or medium. Concerning the effect of the depth-of-cut and the cutting speed, the R^2^ values show weak relationships. In grinding the infeed velocity has the greatest influence on the roughness values. The values of R^2^ vary between 0.005 and 0.269. This means that the relationship is rather weak. Analyzing the multifunctional coefficient of determination, in hard turning it varies between 0.483 and 0.912 and in grinding between 0.457 and 0.842. These show mainly strong or extremely strong relationships.

### 3.3. Connections between the Analyzed Parameters

**Figure 13:**
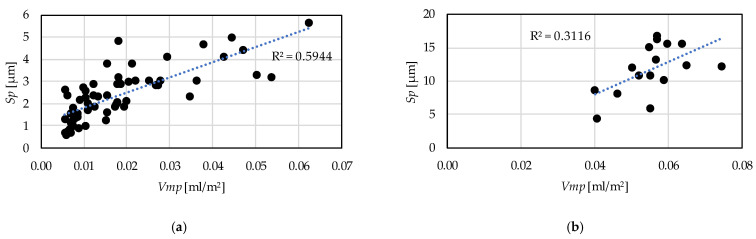
Connection between the peak material volume—*Vmp* and the maximum height—*Sp* parameters (**a**) hard turning; (**b**) grinding.

A strong correlation between the parameters that determine the same functional characteristics of a surface is essential from the point of view of the reliability of the parameters. In Figure 13 the relationship between the *Sp* and the *Vmp* parameters are demonstrated for hard turning and grinding. In the former case the coefficient of determination is 0.59, which indicates a strong relationship. In grinding this value is 0.31, i.e., the strength of the relationship is medium. Two extreme outliers were eliminated in the case of hard turning.

**Figure 14:**
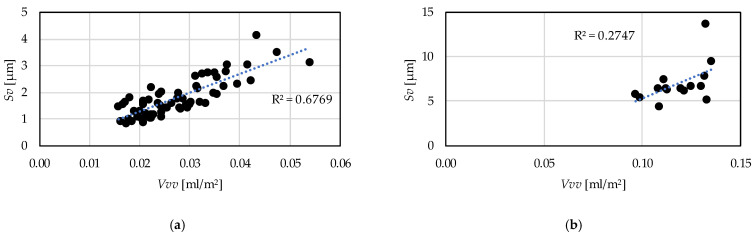
Connection between the valley void volume—*Vvv* and the maximum valley depth—*Sv* parameters (**a**) hard turning; (**b**) grinding.

**Figure 15:**
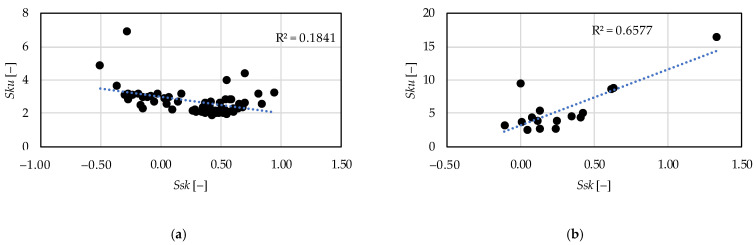
Connection between the skewness—*Ssk* and the kurtosis—*Sku* parameters (**a**) hard turning; (**b**) grinding.

In Figure 14 the relationships between the *Vvv* and the *Sv* parameters are demonstrated. Two extreme outliers were eliminated in the case of hard turning and one in the case of grinding. The coefficient of determination is 0.68 for the hard turning procedure version, which means a strong relationship and 0.27 for grinding, which is medium strength. In Figure 15 the relationships between the skewness (*Ssk*) and the kurtosis (*Sku*) are demonstrated. In hard turning the coefficient of determination is 0.18, which means a medium (but closer to weak) relationship. Furthermore, in grinding the value of this coefficient is 0.66, which means a strong relationship.

From these values of the coefficient of determination it can be stated that in case of hard turning the peak material volume (*Vmp*) and the maximum peak height (*Sp*) values move together; therefore, both are applicable to describe the same property of a surface, such as wear resistance or fluid-retention ability. This is valid for the valley void volume (*Vvv*) and the maximum valley depth (*Sv*). However, in grinding, the relationship between these two parameter counterparts is relatively weak. Concerning the *Ssk* and the *Sku* parameters the opposite was found. The parameters can be considered reliable in the case of grinding.

It can be stated that the medium or strong relationship between some parameters strengthens the expectation that parameter values that quantifying identical surface properties show similar tendencies. At the same time, in the cutting experiments a hardened steel was applied. As the result of the carburizing and case hardening a hard martensitic microstructure was evolved, and it influences the machinability of the material in a relatively high extent. This could explain to the phenomena that the surface topography shows irregularities in contrast to the expectations. These irregularities could result in weaker correlation relationships between the considered parameters.

## 4. Conclusions

Hard turning and grinding experiments were carried out on four parameter levels and, hardened (62–63 HRC) material 16MnCr5 was machined. From the analysis of the topography parameters maximum peak height—*Sp*, maximum valley depth—*Sv*, peak material volume—*Vmp*, valley void volume—*Vvv*, skewness—*Ssk* and kurtosis—*Sku*, the following was found.

- For the prediction of the topography parameters a cubic type response function is applicable. The obtained coefficients of determination (R^2^) varied between 0.48 and 0.91 in the case of hard turning and between 0.46 and 0.84 in the case of grinding.
- The major topography-influencing cutting parameter in hard turning is the feed rate (*f*) and in grinding the infeed velocity (*v*_fR_). The single-factor coefficients of determination vary between 0.2 and 0.65 in hard turning and between 0.05 and 0.27 in grinding. The reason for the relatively low absolute values is the structure of the cubic response function. These values are the highest compared to those of the other components of the response function.
- Based on the hard turning experimental results, there are strong relationships between the peak material volume (*Vmp*) and maximum peak height (*Sp*) parameters (R^2^ = 0.59) and between the valley void volume (*Vvv*) and maximum valley depth (*Sv*) parameters (R^2^ = 0.68). Therefore, both parameters can be reliably used for assessing surface properties. The same is not valid for grinding based on the coefficients of determination 0.31 and 0.27, respectively.
- Based on the grinding experimental results, there is a strong relationship between the skewness (*Ssk*) and the kurtosis (*Sku*) parameters. The coefficient of determination is 0.66. This is not valid for the hard turning (R^2^ = 0.18).

The findings are valid for the machined material and in the analyzed cutting data ranges set based on the tool and workpiece material pair. The study is worth extended in the future to more types of materials, and wear experiments should be carried out to obtain a clearer picture about the connection between the physical wear process and the topography parameters.

## Figures and Tables

**Table 1:** Mechanical and physical properties of the material 16MnCr5 [35].

Tensile Strength [MPa]	Yield Strength [MPa]	Elongation [%]	HRC Hardness	Thermal Conductivity [N/mK]	Specific Heat [J/kgK]	Melting Temperature [°C]
1158	1034	15	62–63	16	500	1370–1400

**Table 2:** Chemical composition of the material 16MnCr5 (DIN EN 10184:2008).

C	Si	Mn	Cr	S	P
0.14–0.19	<0.40	1.00–1.30	0.80–1.10	<0.035	<0.025

**Table 3:** Measured Vickers hardness values of the workpieces.

Measurement	Workpiece							
	A	B	C	D	E	F	G	H
1	800	760	783	743	771	773	766	778
2	788	805	723	741	755	789	778	748
3	776	776	756	766	755	751	766	760
4	741	776	796	737	789	772	801	726
5	784	788	780	769	784	754	754	743

**Table 4:** Experimental setup and the applied cutting data for hard turning.

Setup	Depth-of Cut, *a*_p_ [mm]	Cutting Speed, *v*_c_ [m/min]	Feed Rate, *f* [mm/rev]	Setup	Depth-of Cut, *a*_p_ [mm]	Cutting Speed, *v*_c_ [m/min]	Feed Rate, *f* [mm/rev]	Setup	Depth-of Cut, *a*_p_ [mm]	Cutting Speed, *v*_c_ [m/min]	Feed Rate, *f* [mm/rev]	Setup	Depth-of Cut, *a*_p_ [mm]	Cutting Speed, *v*_c_ [m/min]	Feed Rate, *f* [mm/rev]
T_1_	0.05	60	0.05	T_17_	0.1	60	0.05	T_33_	0.2	60	0.05	T_49_	0.3	60	0.05
T_2_	0.05	60	0.1	T_18_	0.1	60	0.1	T_34_	0.2	60	0.1	T_50_	0.3	60	0.1
T_3_	0.05	60	0.15	T_19_	0.1	60	0.15	T_35_	0.2	60	0.15	T_51_	0.3	60	0.15
T_4_	0.05	60	0.2	T_20_	0.1	60	0.2	T_36_	0.2	60	0.2	T_52_	0.3	60	0.2
T_5_	0.05	90	0.05	T_21_	0.1	90	0.05	T_37_	0.2	90	0.05	T_53_	0.3	90	0.05
T_6_	0.05	90	0.1	T_22_	0.1	90	0.1	T_38_	0.2	90	0.1	T_54_	0.3	90	0.1
T_7_	0.05	90	0.15	T_23_	0.1	90	0.15	T_39_	0.2	90	0.15	T_55_	0.3	90	0.15
T_8_	0.05	90	0.2	T_24_	0.1	90	0.2	T_40_	0.2	90	0.2	T_56_	0.3	90	0.2
T_9_	0.05	120	0.05	T_25_	0.1	120	0.05	T_41_	0.2	120	0.05	T_57_	0.3	120	0.05
T_10_	0.05	120	0.1	T_26_	0.1	120	0.1	T_42_	0.2	120	0.1	T_58_	0.3	120	0.1
T_11_	0.05	120	0.15	T_27_	0.1	120	0.15	T_43_	0.2	120	0.15	T_59_	0.3	120	0.15
T_12_	0.05	120	0.2	T_28_	0.1	120	0.2	T_44_	0.2	120	0.2	T_60_	0.3	120	0.2
T_13_	0.05	150	0.05	T_29_	0.1	150	0.05	T_45_	0.2	150	0.05	T_61_	0.3	150	0.05
T_14_	0.05	150	0.1	T_30_	0.1	150	0.1	T_46_	0.2	150	0.1	T_62_	0.3	150	0.1
T_15_	0.05	150	0.15	T_31_	0.1	150	0.15	T_47_	0.2	150	0.15	T_63_	0.3	150	0.15
T_16_	0.05	150	0.2	T_32_	0.1	150	0.2	T_48_	0.2	150	0.2	T_64_	0.3	150	0.2

**Table 5:** Experimental setup and the applied cutting data for grinding.

Setup	Infeed Velocity *v*_fR_ [mm/s]	rpm *n* [1/min]	Setup	Infeed Velocity *v*_fR_ [mm/s]	rpm *n* [1/min]	Setup	Infeed Velocity *v*_fR_ [mm/s]	rpm *n* [1/min]	Setup	Infeed Velocity *v*_fR_ [mm/s]	rpm *n* [1/min]
G_1_	31.5	0.0069	G_5_	31.5	0.0130	G_9_	31.5	0.0193	G_13_	31.5	0.0302
G_2_	45	0.0069	G_6_	45	0.0130	G_10_	45	0.0193	G_14_	45	0.0302
G_3_	63	0.0069	G_7_	63	0.0130	G_11_	63	0.0193	G_15_	63	0.0302
G_4_	90	0.0069	G_8_	90	0.0130	G_12_	90	0.0193	G_16_	90	0.0302

**Table 6:** Coefficients of determination of the influencing cutting data and the multifactorial coefficients of determination.

Analyzed Parameter		Coefficients of Determination of the Influencing Factors					Multifactorial Coefficients of Determination	
		$R_{HT,\;a_{p}}^{2}$	$R_{HT,\;v_{c}}^{2}$	$R_{HT,\;f}^{2}$	$R_{G,\;v_{fR}}^{2}$	$R_{G,\;n}^{2}$	$R_{HT}^{2}$	$R_{G}^{2}$
*arithmetic mean height*	*Sa*	0.074	0.004	0.643	0.145	0.028	0.912	0.808
*maximum height*	*Sz*	0.078	0.002	0.468	0.121	0.038	0.784	0.787
*peak material volume*	*Vmp*	0.048	0.016	0.639	0.269	0.068	0.875	0.815
*valley void volume*	*Vvv*	0.022	0.018	0.292	0.120	0.121	0.741	0.574
*maximum peak height*	*Sp*	0.061	0.004	0.383	0.082	0.001	0.698	0.842
*maximum valley depth*	*Sv*	0.104	0.001	0.519	0.073	0.196	0.826	0.457
*skewness*	*Ssk*	0.002	0.008	0.650	0.015	0.044	0.764	0.649
*kurtosis*	*Sku*	0.010	0.002	0.201	0.005	0.000	0.483	0.738

## Data Availability

Not applicable.

## Appendix A

**Table A1:** Topography parameter data (hard turning, *a*_p_ = 0.05 mm).

*a*_p_	*v*_c_	*f*	*Sa*	*Sz*	*Vmp*	*Vvv*	*Sp*	*Sv*	*Ssk*	*Sku*
0.05	60	0.05	0.122	1.631	0.007	0.018	0.695	0.936	−0.029	3.148
0.05	60	0.1	0.272	2.041	0.009	0.020	0.937	1.104	0.482	2.129
0.05	60	0.15	0.491	3.424	0.017	0.025	1.986	1.438	0.671	2.395
0.05	60	0.2	0.940	7.023	0.047	0.035	4.430	2.593	0.657	2.396
0.05	90	0.05	0.125	1.802	0.007	0.018	0.898	0.905	0.045	2.545
0.05	90	0.1	0.205	2.466	0.007	0.020	1.154	1.312	−0.148	2.286
0.05	90	0.15	0.439	2.662	0.015	0.028	1.260	1.402	0.705	2.633
0.05	90	0.2	0.639	5.670	0.027	0.037	2.863	2.807	0.847	2.566
0.05	120	0.05	0.149	1.609	0.006	0.018	0.622	0.987	−0.231	3.106
0.05	120	0.1	0.171	3.120	0.007	0.019	1.828	1.292	0.351	2.333
0.05	120	0.15	0.378	3.435	0.012	0.024	1.916	1.519	0.342	2.052
0.05	120	0.2	0.749	4.955	0.018	0.029	2.897	1.758	0.551	1.968
0.05	150	0.05	0.129	1.785	0.006	0.021	0.779	1.006	−0.268	3.192
0.05	150	0.1	0.217	2.504	0.008	0.022	1.437	1.067	0.491	2.647
0.05	150	0.15	0.437	3.491	0.018	0.030	2.076	1.415	0.407	2.169
0.05	150	0.2	0.645	4.730	0.022	0.030	3.082	1.647	0.605	2.116

**Table A2:** Topography parameter data (hard turning, *a*_p_ = 0.1 mm).

*a*_p_	*v*_c_	*f*	*Sa*	*Sz*	*Vmp*	*Vvv*	*Sp*	*Sv*	*Ssk*	*Sku*
0.1	60	0.05	0.113	1.756	0.007	0.021	0.870	0.886	−0.365	3.639
0.1	60	0.1	0.193	2.961	0.010	0.022	1.009	1.185	0.142	2.662
0.1	60	0.15	0.317	3.704	0.020	0.030	2.137	1.567	0.583	2.850
0.1	60	0.2	0.553	4.953	0.020	0.035	3.001	1.952	0.534	2.185
0.1	90	0.05	0.134	2.808	0.006	0.016	1.319	1.490	−0.153	2.936
0.1	90	0.1	0.208	3.301	0.009	0.022	1.566	1.735	−0.050	2.700
0.1	90	0.15	0.266	3.808	0.017	0.024	1.872	1.936	0.579	2.851
0.1	90	0.2	0.478	4.086	0.019	0.032	1.918	2.168	0.649	2.528
0.1	120	0.05	0.114	2.817	0.007	0.021	1.592	1.225	−0.506	4.852
0.1	120	0.1	0.164	3.284	0.011	0.024	2.036	1.248	0.416	2.674
0.1	120	0.15	0.267	3.933	0.013	0.026	2.338	1.595	0.373	2.609
0.1	120	0.2	0.447	4.004	0.035	0.032	2.359	1.645	0.950	3.222
0.1	150	0.05	0.181	2.400	0.008	0.024	1.291	1.109	−0.115	2.965
0.1	150	0.1	0.170	3.639	0.009	0.025	2.200	1.439	−0.268	2.845
0.1	150	0.15	0.291	5.588	0.016	0.027	3.821	1.767	0.536	2.856
0.1	150	0.2	0.446	7.336	0.038	0.031	4.709	2.627	0.814	3.180

**Table A3:** Topography parameter data (hard turning, *a*_p_ = 0.2 mm).

*a*_p_	*v*_c_	*f*	*Sa*	*Sz*	*Vmp*	*Vvv*	*Sp*	*Sv*	*Ssk*	*Sku*
0.2	60	0.05	0.270	1.754	0.007	0.017	0.910	0.844	0.031	2.875
0.2	60	0.1	0.432	2.231	0.007	0.023	1.046	1.185	−0.172	2.525
0.2	60	0.15	0.878	6.130	0.025	0.037	3.074	3.056	0.453	1.983
0.2	60	0.2	1.494	7.644	0.043	0.048	4.138	3.506	0.425	1.896
0.2	90	0.05	0.346	3.987	0.015	0.033	2.387	1.600	0.170	3.175
0.2	90	0.1	0.479	5.526	0.018	0.039	3.200	2.326	0.285	2.246
0.2	90	0.15	0.581	6.704	0.029	0.058	4.123	2.581	0.414	2.100
0.2	90	0.2	0.965	10.049	0.062	0.127	5.659	4.390	0.267	2.163
0.2	120	0.05	0.128	4.332	0.006	0.021	2.673	1.659	−0.283	6.930
0.2	120	0.1	0.195	5.100	0.012	0.022	2.916	2.184	0.406	2.390
0.2	120	0.15	0.837	6.056	0.021	0.031	3.825	2.231	0.522	2.062
0.2	120	0.2	1.412	12.051	0.029	0.043	7.887	4.164	0.524	2.009
0.2	150	0.05	0.333	3.324	0.011	0.021	1.757	1.567	0.093	2.246
0.2	150	0.1	0.115	6.860	0.018	0.028	4.881	1.979	0.554	3.982
0.2	150	0.15	0.667	5.067	0.028	0.035	3.068	1.999	0.702	4.410
0.2	150	0.2	1.046	13.034	0.033	0.054	9.900	3.134	0.556	2.072

**Table A4:** Topography parameter data (hard turning, *a*_p_ = 0.3 mm).

*a*_p_	*v*_c_	*f*	*Sa*	*Sz*	*Vmp*	*Vvv*	*Sp*	*Sv*	*Ssk*	*Sku*
0.3	60	0.05	0.112	3.727	0.010	0.028	2.301	1.426	0.073	2.963
0.3	60	0.1	0.253	3.950	0.012	0.029	2.412	1.538	0.293	2.091
0.3	60	0.15	0.570	5.636	0.019	0.033	2.910	2.726	0.501	2.144
0.3	60	0.2	0.998	7.747	0.044	0.035	5.011	2.735	0.542	2.114
0.3	90	0.05	0.109	1.687	0.006	0.016	0.787	0.900	−0.085	3.051
0.3	90	0.1	0.279	4.575	0.010	0.018	2.769	1.806	0.514	2.293
0.3	90	0.15	0.537	5.005	0.018	0.024	2.987	2.018	0.604	2.263
0.3	90	0.2	1.075	5.969	0.054	0.034	3.210	2.759	0.684	2.356
0.3	120	0.05	0.193	3.418	0.006	0.022	2.385	1.033	−0.304	3.120
0.3	120	0.1	0.305	4.186	0.010	0.024	2.597	1.590	0.372	2.037
0.3	120	0.15	0.569	5.095	0.027	0.037	2.875	2.220	0.451	2.134
0.3	120	0.2	1.036	6.159	0.036	0.041	3.091	3.069	0.481	2.013
0.3	150	0.05	0.144	2.304	0.006	0.017	0.732	1.572	−0.186	3.146
0.3	150	0.1	0.344	3.021	0.008	0.017	1.368	1.653	0.468	2.003
0.3	150	0.15	0.666	3.301	0.016	0.021	1.628	1.673	0.648	2.260
0.3	150	0.2	1.083	5.796	0.050	0.042	3.330	2.466	0.616	2.216

**Table A5:** Topography parameter data for grinding.

*n*	*v*_fR_	*Sa*	*Sz*	*Vmp*	*Vvv*	*Sp*	*Sv*	*Ssk*	*Sku*
31.5	0.0069	1.106	18.463	0.050	0.120	11.987	6.476	0.237	2.670
45	0.0069	0.893	8.669	0.041	0.109	4.293	4.376	0.045	2.557
63	0.0069	1.089	13.879	0.055	0.132	5.958	7.921	0.129	2.720
90	0.0069	0.810	14.270	0.046	0.121	8.075	6.195	−0.110	3.231
31.5	0.0130	0.762	23.286	0.057	0.108	16.868	6.417	0.630	8.785
45	0.0130	0.868	18.399	0.057	0.133	13.186	5.213	0.078	4.281
63	0.0130	0.738	22.698	0.057	0.112	16.272	6.426	1.333	16.387
90	0.0130	0.771	28.778	0.055	0.132	15.079	13.699	−0.003	9.385
31.5	0.0193	0.727	15.550	0.059	0.099	10.070	5.480	0.407	4.342
45	0.0193	0.643	14.403	0.040	0.097	8.619	5.785	0.006	3.629
63	0.0193	0.798	23.092	0.060	0.111	15.636	7.456	0.420	5.103
90	0.0193	0.942	19.109	0.065	0.130	12.377	6.732	0.248	3.865
31.5	0.0302	0.752	22.328	0.064	0.125	15.628	6.699	0.616	8.584
45	0.0302	0.744	22.093	0.055	0.006	10.773	11.320	0.130	5.314
63	0.0302	0.779	17.136	0.052	0.113	10.775	6.361	0.117	3.796
90	0.0302	0.967	21.828	0.075	0.135	12.269	9.558	0.350	4.561

**Table A6:** Coefficients of the response functions for hard turning.

	*Sa*	*Sz*	*Vmp*	*Vvv*	*Sp*	*Sv*	*Ssk*	*Sku*
b_0_	1.37	−6.75	0.04	0.07	−9.61	3.13	−0.77	−1.18
b_1_	−15.53	−90.97	−0.08	0.08	−71.76	−23.91	−8.07	26.96
b_2_	−0.02	0.27	0.00	0.00	0.35	−0.08	0.02	0.19
b_3_	−1.45	110.72	−0.11	−0.34	74.11	24.05	10.36	−73.80
b_11_	120.34	640.51	1.17	2.35	464.84	202.58	36.86	−164.53
b_22_	0.00	0.00	0.00	0.00	0.00	0.00	0.00	0.00
b_33_	37.10	−814.11	1.28	2.10	−602.44	−108.37	−4.12	490.98
b_111_	−252.93	−1237.09	−3.65	−8.35	−889.97	−385.09	−60.37	249.15
b_222_	0.00	0.00	0.00	0.00	0.00	0.00	0.00	0.00
b_333_	−28.29	2269.98	2.15	0.64	1656.12	397.07	−104.12	−1389.92
b_12_	−0.03	−0.04	0.00	−0.01	0.06	−0.11	0.05	0.16
b_13_	3.85	369.31	−0.61	3.02	280.67	99.92	8.09	−80.07
b_23_	−0.05	−0.69	0.00	0.00	−0.36	−0.34	−0.02	0.26
b_112_	0.11	0.16	0.00	0.02	0.13	0.00	−0.06	−0.36
b_113_	−34.96	−1016.36	0.37	−9.17	−771.72	−270.11	42.44	317.60
b_221_	0.00	0.00	0.00	0.00	0.00	0.00	0.00	0.00
b_223_	0.00	0.00	0.00	0.00	0.00	0.00	0.00	0.00
b_331_	57.43	47.81	4.90	3.49	71.17	−32.99	−117.26	63.09
b_332_	−0.10	0.80	−0.01	−0.02	0.56	0.07	0.29	0.86
b_123_	0.07	0.05	0.00	0.00	−0.16	0.21	−0.01	−0.53

**Table A7:** Coefficients of the response functions for grinding.

	*Sa*	*Sz*	*Vmp*	*Vvv*	*Sp*	*Sv*	*Ssk*	*Sku*
b_0_	2.74	47.76	0.11	0.40	49.22	−1.46	3.99	−0.66
b_1_	−0.05	−3.88	−0.01	−0.02	−3.90	0.02	−0.37	−2.24
b_2_	−170.01	8045.68	8.75	15.73	6467.88	1577.78	535.11	8856.34
b_11_	0.00	0.06	0.00	0.00	0.06	0.00	0.01	0.04
b_22_	7462.72	−561,230.92	−346.53	−512.09	−426,625.61	−134,604.82	−26,412.82	−506,380.38
b_111_	0.00	0.00	0.00	0.00	0.00	0.00	0.00	0.00
b_222_	−92,984.08	10,903,134.74	6396.40	8736.53	7,982,419.09	2,920,707.30	476,447.27	9,328,540.86
b_12_	0.32	50.70	−0.10	−0.34	31.19	19.51	−3.33	−14.85
b_112_	0.01	−0.12	0.00	0.00	−0.07	−0.05	0.03	0.19
b_122_	−24.57	−995.25	−0.34	0.04	−609.55	−385.70	−17.46	−338.71
